# Decrease of 5-hydroxymethylcytosine and TET1 with nuclear exclusion of TET2 in small intestinal neuroendocrine tumors

**DOI:** 10.1186/s12885-018-4579-z

**Published:** 2018-07-25

**Authors:** Elham Barazeghi, Surendra Prabhawa, Olov Norlén, Per Hellman, Peter Stålberg, Gunnar Westin

**Affiliations:** Department of Surgical Sciences, Uppsala University, Uppsala University Hospital, Rudbeck Laboratory, SE-751 85 Uppsala, Sweden

**Keywords:** 5-hydroxymethylcytosine, TET1, TET2, Epigenetic, Neuroendocrine tumors, SI-NET

## Abstract

**Background:**

Small intestinal neuroendocrine tumors (SI-NETs) originate from enterochromaffin cells scattered in the intestinal mucosa of the ileum and jejunum. Loss of one copy of chromosome 18 is the most frequent observed aberration in primary tumors and metastases. The aim of this study was to investigate possible involvement of 5-hydroxymethylcytosine (5hmC), TET1 and TET2 in SI-NETs.

**Methods:**

The analysis was conducted using 40 primary tumors and corresponding 47 metastases. The level of 5hmC, TET1 and TET2 was analyzed by DNA immune-dot blot assay and immunohistochemistry. Other methods included a colony forming assay, western blotting analysis, and quantitative bisulfite pyrosequencing analysis. The effect of the exportin-1 nuclear transport machinery inhibitors on cell proliferation and apoptosis was also explored using two SI-NET cell lines.

**Results:**

Variable levels of 5hmC and a mosaic staining appearance with a mixture of positive and negative cell nuclei, regardless of cell number and staining strength, was observed overall both in primary tumors and metastases. Similarly aberrant staining pattern was observed for TET1 and TET2. In a number of tumors (15/32) mosaic pattern together with areas of negative staining was also observed for TET1. Abolished expression of TET1 in the tumors did not seem to involve hypermethylation of the TET1 promoter region. Overexpression of TET1 in a colony forming assay supported a function as cell growth regulator. In contrast to 5hmC and TET1, TET2 was also observed in the cytoplasm of all the analyzed SI-NETs regardless of nuclear localization. Treatment of CNDT2.5 and KRJ-I cells with the exportin-1 (XPO1/CRM1) inhibitor, leptomycin B, induced reduction in the cytoplasm and nuclear retention of TET2. Aberrant partitioning of TET2 from the nucleus to the cytoplasm seemed therefore to involve the exportin-1 nuclear transport machinery. Reduced cell proliferation and induction of apoptosis were observed after treatment of CNDT2.5 and KRJ-I cells with leptomycin B or KPT-330 (selinexor).

**Conclusions:**

SI-NETs are epigenetically dysregulated at the level of 5-hydroxymethylcytosine/ TET1/TET2. We suggest that KPT-330/selinexor or future developments should be considered and evaluated for single treatment of patients with SI-NET disease and also in combinations with somatostatin analogues, peptide receptor radiotherapy, or everolimus.

**Electronic supplementary material:**

The online version of this article (10.1186/s12885-018-4579-z) contains supplementary material, which is available to authorized users.

## Background

Small intestinal neuroendocrine tumor (SI-NET) is the most common malignancy of the small intestine and the incidence is about one per 100,000 and is on the rise. These tumors, formerly known as midgut carcinoids, are small with relatively low cellular proliferation (Ki67 proliferation index is often < 2%), although the majority of patients have tumor spread to both lymph nodes and liver at the time of diagnosis with 5-year overall survival rate of around 65%. SI-NETs are also characterized by secreting hormones such as serotonin and tachykinins, which can lead to carcinoid syndrome as a major cause of mortality in these patients. Surgical resection of the primary tumor and metastases is the only curative treatment and may alleviate symptoms caused by small intestinal obstruction or ischaemia and the carcinoid syndrome. Somatostatin analogues used for treatment can improve symptoms, increase time to progression and reduce hormone related morbidity [1].

The knowledge about genetic and epigenetic aberrations in SI-NETs is limited [1]. The most common genetic aberration in these tumors is loss of one copy of chromosome 18, known to occur in > 60% of tumors [2, 3]. Massively parallel whole-exome sequencing of SI-NETs has revealed no recurrent mutations and a generally low rate of mutations, indicating that these tumors may be genetically stable [4]. However, in a study of 180 tumors [5], frame shift mutations (7.8%) and hemizygous deletions (14%) were found in *CDKN1B* gene, which encodes the cyclin-dependent kinase inhibitor p27. Pathogenic mutations of *CDKN1B* was corroborated in a further study of 362 tumors from 200 patients (8.5%) [6]. Promoter hypermethylation of *RASSF1A*, *CTNNB1* and several other genes have been reported [7–10]. *TCEB3C* encodes elongin A3 is located at 18q21, and has been shown to be epigenetically repressed and has a growth regulatory role in SI-NET cells [11].

The ten-eleven translocation (TET) protein family was discovered in 2009 [12] to have the capacity of catalyzing the conversion of 5-methylcytosine to 5-hydroxymethylcytosine (5hmC) and further to 5-formyl- and 5-carboxylcytosine, to not only generate new epigenetic marks, but also initiate active or passive demethylation pathways [13]. Recent studies have shown that aberrant expression of TETs and 5hmC levels are associated with tumorigenesis in different types of cancers [14–19], and TET2 is frequently mutated in hematological malignancies [20, 21]. In this study, we investigate the level of 5hmC and expression of TET1 and TET2, and also present data demonstrating loss of nuclear localization of TET2 in SI-NETs, for the first time. Potential treatment of patients with SI-NET disease with inhibitors of nuclear export is discussed.

## Methods

### Tissue specimens

All 40 patients included in this study (Additional file 1: Table S1) were diagnosed with SI-NET and operated on at Uppsala University Hospital between 2000 and 2016. The mean age at diagnosis was 62.5 years (range 42–79 years). Specimens were stained with hematoxylin-eosin and those with apparent tumor mass were selected for analyses. In total 87 tumors were analyzed; 40 primary tumors, 37 mesometastases, 7 liver metastases, and 3 lymph node metastases, as well as 4 “normal” small intestine tissue specimens. In addition to being embedded in paraffin, all tissue specimens were snap frozen in liquid nitrogen and kept at − 70 °C. Two human SI-NET cell lines were used in the experiments. CNDT2.5 adhesive cells developed from a liver metastases from a patient diagnosed with primary ileal SI-NET, were kindly provided by Dr. Lee Ellis, MD, Anderson Cancer Center, Houston, TX, USA, and used in this study at cell passages 10–30 [22, 23]. KRJ-I suspension cells established from a multifocal metastatic ileal carcinoid tumor were a kind gift from Dr. Roswitha Pfragner, Medical University of Graz, Austria [24–26]. Both cell lines expressed the neuroendocrine cell marker synaptophysin (Additional file 2: Figure S1a). In contrast to a recently published study suggesting lymphoblastoid origin of KRJ-I cells [27], the KRJ-I cells used here stained negatively for the lymphoid marker CD45 and the T-cell marker CD3 (Additional file 2: Figure S1b).

### DNA immune-dot blot assay

Genomic DNA was extracted from 87 frozen primary tumors and the matched metastases from 40 patients using DNeasy Blood and tissue kit (Qiagen GmbH) according to the manufacturer’s instructions. 5hmC DNA standard (Zymo Research Corporation) was used as a control. One microgram DNA was denatured in 0.1 M NaOH at 95 °C for 10 min, then neutralized with 1 M ammonium acetate on ice. Twofold serial dilutions of the DNA samples were spotted onto Hybond-N+ nylon membrane (GE Healthcare) in a Bio-Dot apparatus (Bio-Rad Laboratories, Inc.), which was fixed with UV irradiation (GS Gene Linker UV chamber, Bio-Rad). Subsequently, the membrane was blocked with 5% skimmed milk, and incubated at 4 °C overnight with a rabbit polyclonal anti-5hmC antibody (1:10 K dilution, 39,791; Active Motif). After incubation with an appropriate HRP-conjugated secondary antibody, signals were visualized with the enhanced chemiluminescence system (GE Healthcare). To ensure equal amount of the total DNA on the membrane, the same membrane was stained with 0.02% methylene blue in 0.3 M sodium acetate. The second dot blot signal from the top for each serial dilution was used to quantify the 5hmC relative intensity by the NIH Image-J software according to the program’s instructions using a procedure described previously [18].

### Immunohistochemistry

Paraffin embedded tumor tissue sections were deparaffinized with xylene and rehydrated through descending alcohol concentrations and distilled water. Background staining was blocked with 3% hydrogen peroxide and heated in EDTA pH 8.0 (Life Technologies Corporation), for 40 min with microwave at 300-W power. Then the sections were incubated with 2 M HCl for 2.5 min and treated with normal goat serum and the rabbit polyclonal anti-5hmC antibody (1:6000 dilution, Active Motif), or the rabbit polyclonal anti-TET1 (HPA019032; Prestige Antibodies, Sigma-Aldrich). For TET2 staining, the sections were heated in citrate buffer pH 6.0 for 10 min with microwave at 800-W power and then for 20 min at 450-W power. After 20 min rest, the sections were incubated with normal goat serum and the rabbit polyclonal anti-TET2 (21207–1-AP, Proteintech Group). For XPO1 staining, the sections were heated in Tris-EDTA buffer pH 9.0 (Bethyl IHC-101 J) and then incubated with normal goat serum and the rabbit monoclonal anti-XPO1 (ab191081, Abcam). The sections were washed three times with PBS, then incubated with a proper secondary antibody and ABC complex. DAB was used for visualization. Consecutive tissue sections of intestinal mucosa were stained with the anti-5hmC antibody (Active Motif), anti-chromogranin A antibody (LK2H10, Thermo Fisher Scientific), anti-TET1 (HPA019032; Prestige Antibodies, Sigma-Aldrich), or anti-TET2 (21207–1-AP, Proteintech). CNDT2.5 and KRJ-I cells first were fixed in formalin and incubated for 20 min with ice-cold 70% ethanol. Then the slides were stained for TET2 as described above. Formalin-fixed CNDT2.5 and KRJ-I cells were first heated in citrate buffer pH 6.0, then incubated with normal goat serum and the rabbit monoclonal anti-synaptophysin (ab32127, Abcam). KRJ-I cells were also stained with the mouse monoclonal anti-CD45 (sc-1178, Santa Cruz Biotechnology), and the rabbit monoclonal anti-CD3 (ab16669, Abcam).

### Cell transfection and drug treatment

CNDT2.5 cells were distributed onto six-well plates (2 × 10^5^) in DMEM-F12 complemented with 10% fetal bovine serum (Sigma Aldrich), 1% nonessential amino acids, and 1% penicillin-streptomycin (PEST), and were cultured at 37 °C in 5% CO2. After overnight incubation, the cells were transfected in triplicate with 4 μg TET1 plasmid expression vector (InvivoGen) or empty vector (pUNO1) using 8 μl Lipofectamin 2000 transfection reagent (Life Technologies), according to the manufacturer’s instructions. Six hours after transfection, a fresh medium was added complemented with 8 μg/ml blasticidin (InvivoGen). KRJ-I cells were seeded onto twelve-well plates (1 × 10^5^) prior to transfection in DMEM-F12 complemented with 10% fetal bovine serum (Sigma Aldrich), 1% nonessential amino acids, and 1% penicillin-streptomycin. The cells were transfected with 0.4 μg TET1 plasmid or empty vector using 1.5 μl Attractene transfection reagent (Qiagen) according to the manufacturer’s instructions. Successful transfection were monitored by RT-PCR and western blotting analysis after 72 h.

CNDT2.5 (2 × 10^5^) and KRJ-I (1 × 10^5^) cells were treated with 5-aza-2′-deoxycytidine (10 μM and 5 μM, respectively) for 72 h. Growth medium with additions were changed every 24 h. Both cell lines were treated with several concentrations with leptomycin B (sc-202,210, Santa Cruz Biotechnology), the nuclear export inhibitor, or KPT-330/selinexor (Selleckchem), an orally bioavailable selective inhibitor of nuclear export for 24 and 72 h, respectively. Two biological replicates of the experiments were performed.

### Colony forming assay, cell proliferation and apoptosis

CNDT2.5 cells were seeded onto six-well plates (2 × 10^5^) and transfected in triplicates with TET1 plasmid expression vector or empty vector as described above. Twenty-four hours after transfection, 2000 cells were distributed onto six-well plates, a fresh medium with 8 μg/ml blasticidin was added every 72 h. After 10 days in blasticidin selection, the cells were fixed with 10% acetic acid/10% methanol and stained with 0.4% crystal violet, and the visible colonies were counted. Three biological replicates of the experiment were performed.

To assess cell proliferation, the CyQUANT cell proliferation assay kit (Invitrogen, Thermo Fisher Scientific) was used according to manufacturer’s instructions. The cells were first frozen in the microplate and then lysed and stained with CyQuant GR dye solution. Infinite 200 PRO (TECAN) plate reader was used to measure fluorescence intensity at 480/520 nm. Apoptosis was measured using the Cell Death Detection ELISA kit (Roche Molecular Biochemicals), according to manufacturer’s protocol. As a positive control, CNDT2.5 and KRJ-I cells were incubated with 0.1 μg/ml camptothecin (Sigma-Aldrich) for 48 h.

### Pyrosequencing

TET1 promoter methylation status was investigated for 12 CpG sites in 20 primary tumors and metastases, CNDT2.5 and KRJ-I cells. DNA was treated with bisulfite using the EpiTect bisulfite kit (Qiagen) according to the manufacturer’s instructions, and PCR amplified using MyTaq™ HS Mix (Bioline). Touchdown PCR was performed with initial denaturation at 95 °C for 2 min, followed by 10 cycles of 95 °C, 15 s; annealing temperature step downs every cycle of 0.5 °C (from 61 °C to 57 °C); 72 °C, 20 s; the annealing temperature for the final 40 cycles was 57 °C followed by denaturation and extension phases as above. Forward primer was used: 5′-GGGGTTAGGGTTTTGATTGTGTTG-3′, and reverse primer: 5′-biotin-ACCCCCAACTCCAAACCTA-3′. Sequencing primer: 5′-GGTTTTGATTGTGTTGGGAG-3′, and sequenced analyzed: 5′- TYGGTAGGYGTTTTTYGYGATTYGTTYGYGTTTTTYGYGTTYGTYG.

GGGTTTYGGGTTTTAAAGTTGTGGGGAT-3′. The pyrosequencing was carried out using 10 μl PCR product with the PyroMark Q24 system (Qiagen) according to manufacturer’s instructions.

### Western blotting analysis

Cytobuster Protein Extract Reagent (Merck Millipore, Billerica, MA, USA) complemented with protease inhibitor cocktail (Roche Diagnostics Scandinavia AB, Bromma, Sweden) used to prepare protein extracts. Rabbit polyclonal anti-TET1 (GTX124207, GeneTex Inc) and goat polyclonal anti-Actin (sc-1616, Santa Cruz Biotechnology) were used. Separate cytoplasmic and nuclear protein extracts were prepared using NE-PER Nuclear and Cytoplasmic Extraction Reagents kit (Thermo Scientific) with Halt Protease Inhibitor Single-Use Cocktail EDTA-free (Thermo Scientific) according to manufacturer’s instructions. Rabbit polyclonal anti-TET2 (21207–1-AP, Proteintech Group), mouse monoclonal anti-Lamin A/C (sc-376,248, Santa Cruz Biotechnology), and rabbit polyclonal anti-β tubulin (sc-9104, Santa Cruz Biotechnology) were used. After incubation with the proper secondary antibody, except for anti-Lamin A/C-HRP conjugated, bands were visualized using the enhanced chemiluminescence system (GE Healthcare).

### Quantitative real time RT-PCR

DNA-free total RNA was extracted using RNeasy Plus Mini kit (Qiagen) according to manufacturer’s instructions and treated with DNase I using TURBO DNA-free™ kit (Life Technologies Corporation, Thermo Fisher Scientific), and PCR used to establish successful treatment of all RNA preparations. cDNA synthesis was performed with random hexamer primers using the First-Strand cDNA Synthesis kit (Fermentas, Thermo Fisher Scientific) according to the manufacturer’s instructions. qRT-PCR was performed on StepOnePlus RealTime PCR systems (Life Technologies Corporation) using TaqMan gene expression Master Mix and assays for TET1 (Hs00286756_m1), TET2 (Hs00325999_m1), XPO1 (Hs00185645_m1), 18S rRNA (Hs03928990_g1), and GAPDH (Hs02758991_g1) transcripts. Each cDNA sample was analyzed in triplicates.

### Statistical analysis

Paired and unpaired *t* test were used for statistical analysis, and *p* < 0.05 was considered significant. All data are presented as mean ± S.D.

## Results

### Overall variable levels and mosaic appearance of the epigenetic mark 5hmC in SI-NETs

**Fig. 1:**
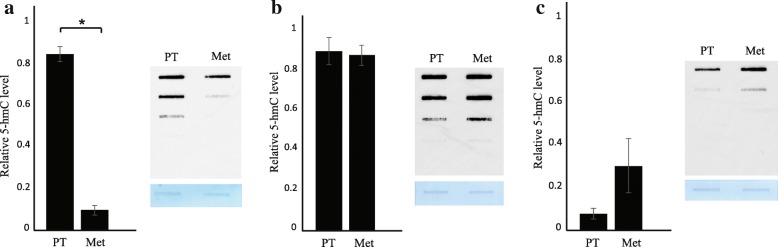
Relative levels of 5hmC by DNA immune-dot blot analysis in SI-NETs from 40 patients separate into 3 groups. **a**: Low level of 5hmC in metastases compared to primary tumors (*n* = 17). **b**: No difference in 5hmC levels (*n* = 15). **c**: High level of 5hmC in metastases compared to primary tumors (*n* = 8). *, *p* < 0.05 (paired *t* test)

The relative level of 5hmC in 87 primary tumors (PT) and corresponding metastases (Met) from 40 patients was determined by a semi-quantitative DNA immune-dot blot assay using a specific rabbit polyclonal antibody against 5hmC [18]. A commercial 5hmC DNA ELISA kit was also applied, but showed too low sensitivity (not shown). The obtained data separated the 40 patients into 3 groups (Fig. 1a - c). Seventeen patients showed a relative low level of 5hmC in the Mets compared to the PTs, 15 patients showed no difference, and 8 patients showed a relative high level of 5hmC in the Mets compared to the PTs. Statistical analysis did not reveal any relation to clinical data (Additional file 1: Table S1) for the 3 patient groups (not shown).

**Fig. 2:**
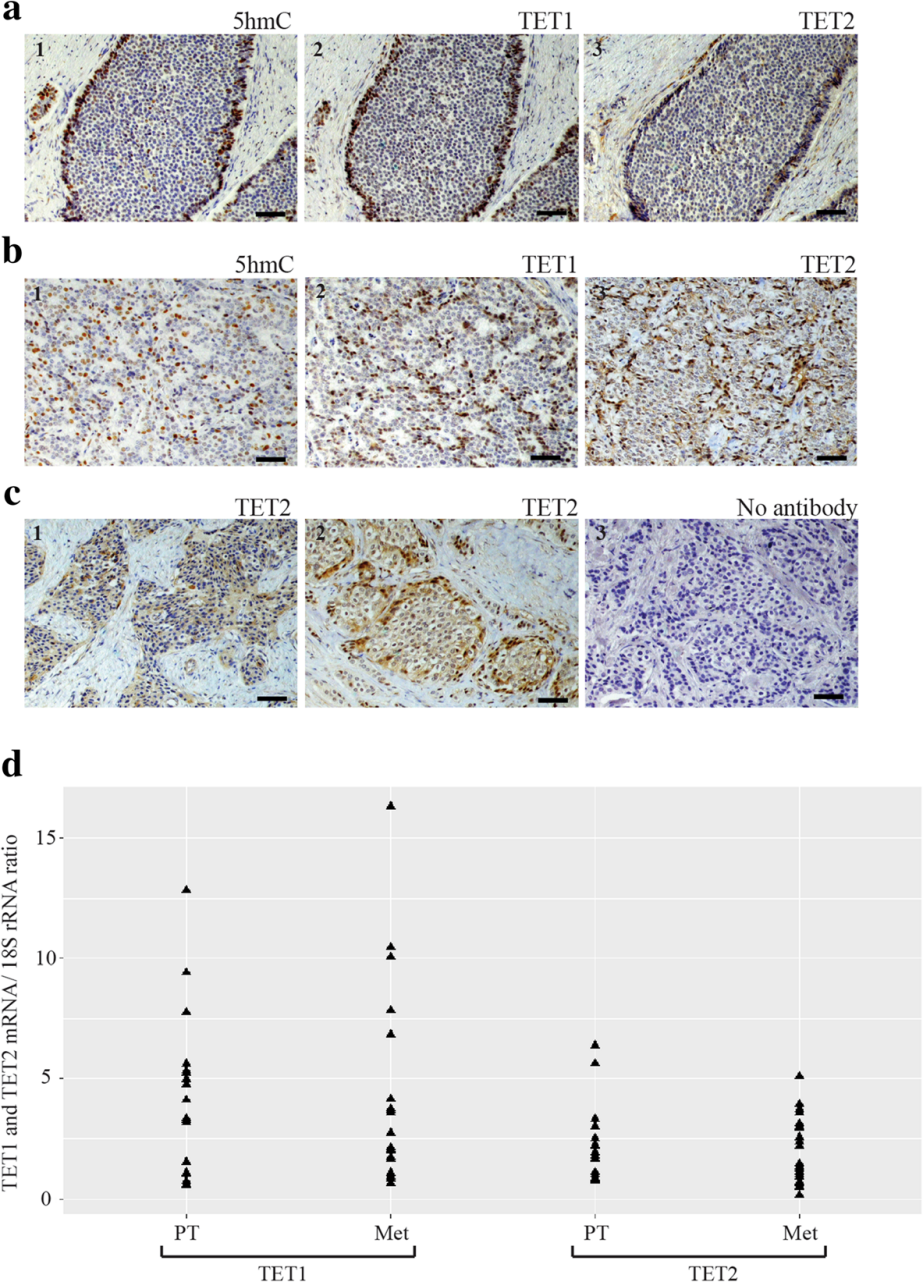
IHC analysis of 5hmC, TET1, and TET2. Representative results are shown. Scale bar, 50 μm. **a**: A primary SI-NET shows a large area with negatively stained cell nuclei for; (1) 5hmC, (2) TET1, (3) TET2. **b**: A metastatic tumor shows mosaic pattern of staining, i.e. a mixture of positive and negative cell nuclei, regardless of cell number, staining strength for; (1) 5hmC, (2) TET1, (3) TET2 with Cytoplasmic staining. **c**: Two primary SI-NETs with (1) the mosaic TET2 cytoplasmic staining together with the presence of negative nuclei, (2) the cytoplasmic staining together with the mosaic nuclear staining patterns, (3) Negative staining was observed only without the primary antibody. **d**: Real-time RT-PCR of TET1 and TET2 reveals no difference in expression between paired primary tumors (*n* = 19) and metastases (*n* = 22)

Next, immunohistochemical (IHC) analysis of 5hmC was performed in 32 paired PTs and Mets from 15 patients, represented in the 3 identified patient groups above (Fig. 1). All analyzed SI-NETs showed a mosaic pattern of staining, i.e. a mixture of positive and negative cell nuclei, regardless of cell number, staining strength or patient group (Fig. 2a, b). Some tumors (9/32) also displayed mosaic pattern together with negative 5hmC staining in larger areas of the section (Fig. 2a). 11 out of 15 patients displayed similar pattern of staining for both primary tumors and metastases and 4 patients displayed mosaic pattern together with negative 5hmC staining in larger areas of the section only in metastases. No staining was observed in the absence of the primary antibody (Fig. 2c), and chromogranin A-positive cells in the normal small intestine stained positive for 5hmC (Additional file 3: Figure S2). These cells likely represent the enterochromaffin cell of origin of SI-NETs.

### Aberrant expression of TET1 and TET2 in SI-NETs

IHC staining of TET1 and TET2 in the 32 paired PTs and Mets from 15 patients closely resembled those observed for 5hmC, with cell nuclear staining and mosaic pattern, i.e. a mixture of positive and negative cell nuclei, regardless of cell number, staining strength or patient group (Fig. 2a-c). Similar to the observed aberrant staining patterns of 5hmC, tumors displayed mosaic regions together with areas of negative staining (15 out of 32 tumors for TET1, and 2 out of 32 tumors for TET2). For one additional tumor almost all cell nuclei stained positive for TET1. Prominent overall staining of TET1 and TET2 was observed in normal small intestine (Additional file 3: Figure S2).

TET2 also showed cytoplasmic staining in addition to the mosaic nuclear staining pattern (Fig. 2a - c). Fifteen out of the analyzed 32 SI-NETs showed cells with TET2 staining of the cytoplasm and of the nucleus. Seventeen tumors showed the presence of cells with detectable TET2 staining of the cytoplasm and not in the nucleus (Fig. 2c). Different patterns of staining in primary tumors compared to paired metastases were observed in 7 patients out of 15 for TET1, and in 2 patients for TET2. The observed staining patterns of TET1 resembled those of 5hmC in 19 tumors out of 32 and for 3 tumors staining of consecutive tissue sections revealed the same negative areas for both 5hmC and TET1. A correlation between the TET1 and TET2 stainings and the Ki67 index (Additional file 1: Table S1) was not observed. No significant difference in mRNA expression level of TET1 and TET2 was observed when PTs and Mets were compared (Fig. 2d).

### Down-regulation of TET1 expression in SI-NETs is not due to promoter hypermethylation

Treatment of SI-NET cell lines CNDT2.5 (adherent cells) and KRJ-I (suspension cells) with the DNA methylation inhibitor 5-aza-2′-deoxycytidine caused clear induction of TET1 mRNA expression in the CNDT2.5 cells. No effects on TET2 expression was observed (Additional file 4: Figure S3a). The TET1 promoter and exon 1 CpG island has been reported to be methylated in multiple cancers [28]. Therefore, DNA methylation levels of 12 CpG residues in this region was determined for the 2 cell lines by quantitative bisulfite pyrosequencing analysis. CNDT2.5 cells showed high level of methylation (90%), while KRJ-I cells were methylated at low level (20%) (Additional file 4: Figure S3b). This was consistent with the observed activation of TET1 expression in Aza-treated CNDT2.5 cells. Next, methylation levels were determined in 20 PTs and Mets, and only very low levels of methylation (10%) were observed (Additional file 4: Figure S3b). Thus, it is not likely that the observed reduced expression of TET1 in SI-NETs involves DNA hypermethylation of the promoter region.

### Growth regulatory role of TET1 in SI-NET cells

**Fig. 3:**
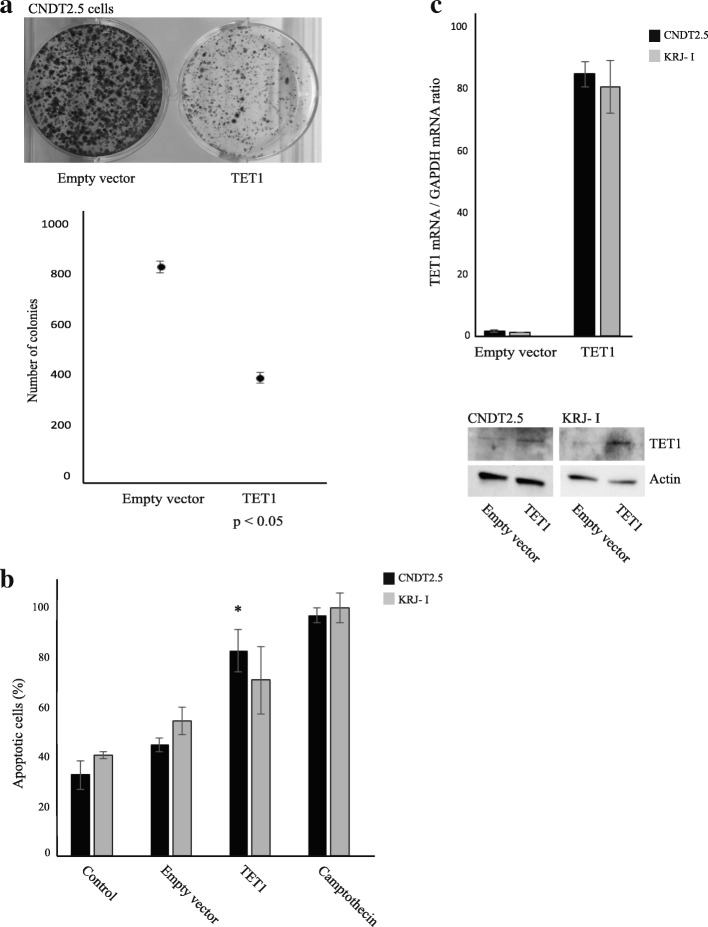
Growth regulatory role of TET1 in SI-NET cells. **a** Colony formation assay in the adhesive SI-NET cell line CNDT2.5. Cells transfected with a TET1 expression vector or empty vector were selected for blasticidin resistance in 10 days and then counted. A representative result is shown and below quantification of triplicate is shown. **b** Apoptosis was analyzed by quantifying cytoplasmic histone-associated-DNA-fragments 72 h after transient transfection. Incubation in 0.1 μg/ml camptothecin was used as positive control. *, *p* < 0.05. **c** TET1 expression at the mRNA and protein level was increased after transfection of the TET1 expression vector

In order to investigate whether TET1 could play a cell growth regulatory role in SI-NET cells, a colony forming assay was used. Stable overexpression of TET1 for 10 days resulted in a significant reduced number of CNDT2.5 cell colonies, compared to transfection of empty expression vector (Fig. 3a). Transient overexpression of TET1 for 72 h in CNDT2.5 and KRJ-I cells resulted in induction of apoptosis, as determined by quantifying cytoplasmic histone-associated-DNA-fragments (Fig. 3b). Successful increased expression of TET1 after transfection was monitored by real-time quantitative PCR and Western blotting analysis (Fig. 3c). Neither transfection with TET2 CRISPR double nickase plasmids [29] nor TET2 shRNAs resulted in efficient knockout of TET2 expression (data not shown).

Taken together, these results strongly suggest a cell growth regulatory role of TET1 in neuroendocrine cells of the small intestine and function as candidate tumor suppressor gene.

### Inhibitors of nuclear export cause nuclear accumulation of TET2, reduced cell proliferation, and induction of apoptosis

**Fig. 4:**
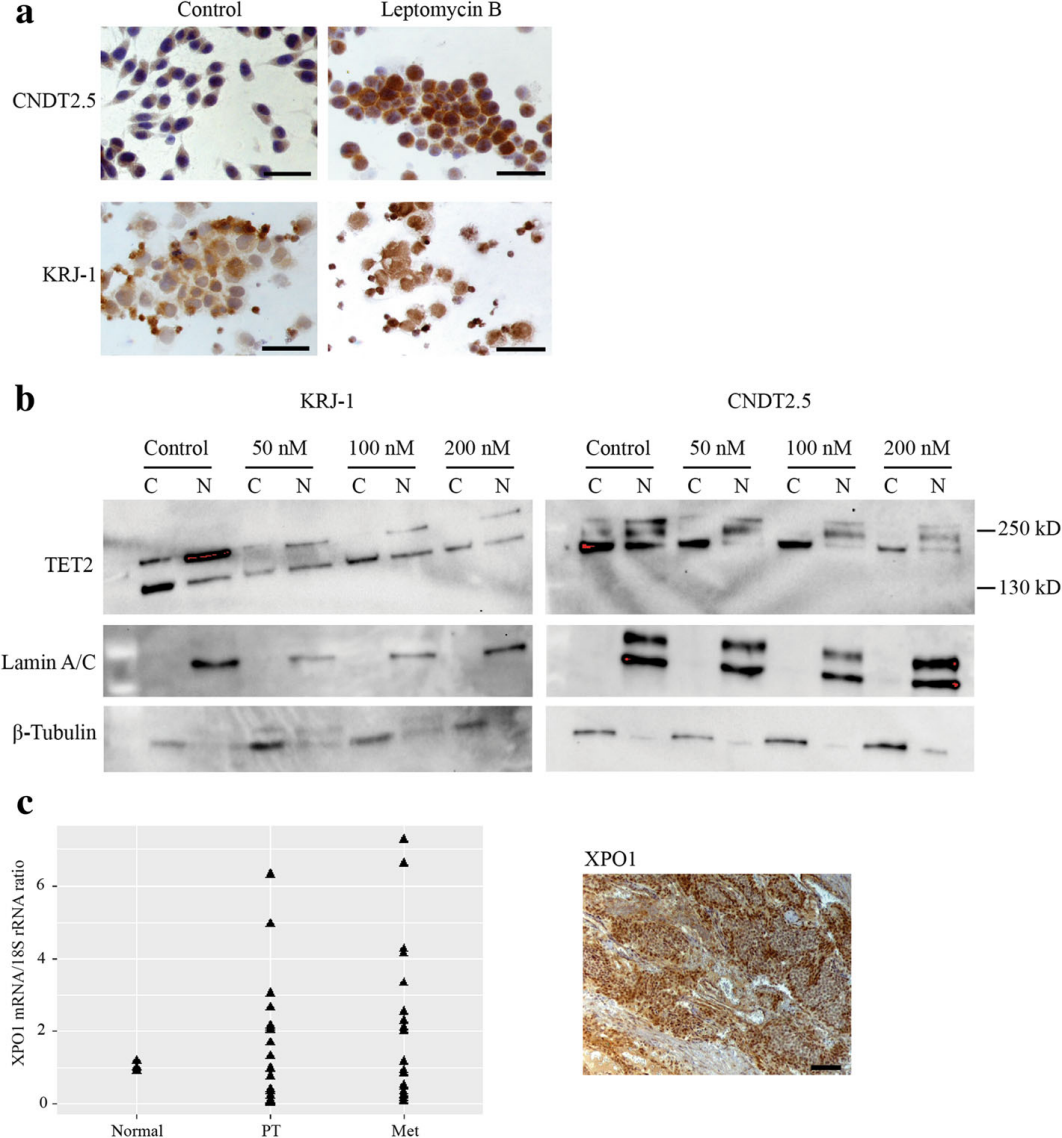
Inhibition of nuclear export causes loss in the cytoplasm and accumulation in the nucleus of TET2. **a** IHC analysis of TET2 after treatment of the SI-NET cell lines CNDT2.5 (adhesive cells) and KRJ-I (suspension cells) with the exportin-1 (XPO1/CRM1) inhibitor leptomycin B. Scale bar, 50 μm. Nuclear accumulation of TET2 is seen. Small labeled dots in the treated KRJ-I cells constitute cellular debris. **b** Western blot analysis of cytoplasmic and nuclear protein extracts from CNDT2.5 and KRJ-I cells after leptomycin B treatment. Induced absence in the cytoplasm and nuclear retention of TET2 is seen. β-tubulin and lamin A/C was used as marker for the cytoplasm and nucleus, respectively. **c** Real-time RT-PCR of XPO1 in paired primary SI-NETs (*n* = 19) and metastases (*n* = 22). Normal tissues from the small intestine (*n* = 3) was arbitrarily used for comparison. A representative overall positively XPO1 stained metastatic tumor is shown to the right. Scale bar, 100 μm

**Fig. 5:**
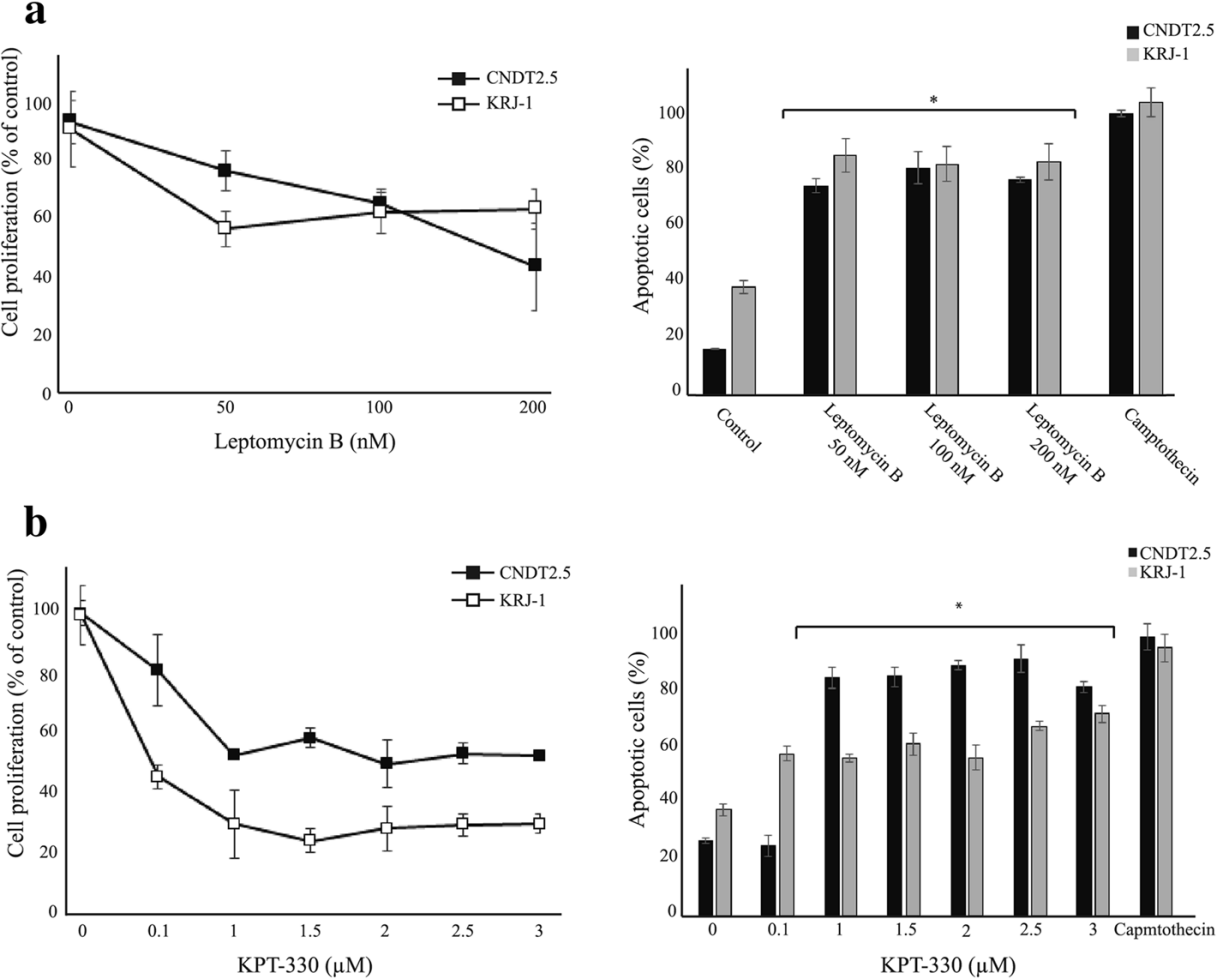
Inhibitors of nuclear export reduce cell proliferation and induce apoptosis of SI-NET cell lines CNDT2.5 and KRJ-I. **a** Treatments with leptomycin B. **b** Treatments with KPT-330/selinexor. *, *p* < 0.05

In addition to the mosaic nuclear staining pattern, the IHC analysis described above displayed variable cytoplasmic expression of TET2 in all analyzed PTs and Mets (*n* = 32), and presence of cytoplasmic positive cells with absent TET2 nuclear localization in 17 of these tumors. To investigate whether the nuclear exclusion of TET2 in SI-NETs involved the nuclear export machinery and the nuclear export protein exportin-1 (XPO1/CRM1), CNDT2.5 and KRJ-I cells were treated with the nuclear export inhibitor leptomycin B for 24 h, and analyzed by IHC and Western blotting. Figure 4a shows accumulation of TET2 in the nuclei of leptomycin B treated cells from both SI-NET cell lines. Partitioning of cellular protein extracts into cytoplasmic and nuclear fractions followed by Western blotting analysis showed induced absence in the cytoplasm and nuclear retention of the slowest migrating polypeptide of TET2 after leptomycin B treatment (Fig. 4b). These results together strongly suggested aberrant transport of TET2 from the nucleus to the cytoplasm by the exportin-1 nuclear export machinery. Overexpression of XPO1 is common in other tumor types and real-time quantitative RT-PCR analysis revealed highly variable mRNA expression levels (Fig. 4c), suggesting a possibility of XPO1 overactivity also in SI-NETs. IHC staining of XPO1 in the 31 PTs and Mets from 15 patients showed overall positive staining regardless of strength in 13 tumors, and mosaic pattern of staining or positive staining together with negative areas was observed in 18 tumors (data not shown). Of the 13 tumors that stained overall positively for XPO1, 12 tumors showed detectable TET2 staining in the cytoplasm and not in the nucleus. Furthermore, treatment with leptomycin B or KPT-330 (selinexor), a selective oral reversible inhibitor of XPO1 and nuclear export [30], resulted in reduced cell proliferation and induction of apoptosis of CNDT2.5 and KRJ-I cells (Fig. 5a, b).

## Discussion

In a wide variety of human cancers, reduced expression of TET1 and TET2 proteins and loss of 5hmC has been documented, and the tumor suppressive role of TET proteins is extensively associated with the prevention of tumorigenesis. Recently, we have shown that in parathyroid carcinomas undetectable/low levels of 5hmC is associated with aberrant expression of TET1 and TET2 and that both genes have cell growth regulatory roles in parathyroid tumor cells [29]. In the present study, overall variable levels of 5hmC and mosaic appearance of 5hmC, TET1, and TET2 were observed in SI-NETs, and a growth regulatory role of TET1 in SI-NET cells in vitro could be demonstrated. For TET2 we failed to obtain efficient knockout of expression for unknown reasons. In contrast to parathyroid tumor cells [29], TET2 is perhaps indispensable for SI-NET cells.

For TET2, expression was also observed in the cytoplasm regardless of detectable expression in the nucleus. Mislocalization to the cytoplasm is expected to cause functional inactivation of TET2. We found that regulation of TET2 localization seemed to involve the exportin-1 nuclear export machinery as treatment of SI-NET cells with the inhibitor of exportin-1, leptomycin B, caused vanished levels in the cytoplasm of the slower migrating TET2 polypeptide, and not of the more faster migrating one.

Loss of nuclear localization of TET2 has also been reported to occur in colorectal cancer and of TET1 in gliomas [31, 32]. Cytoplasmic expression of TET1 was not detected in the SI-NETs analyzed here and loss of TET1 expression could not be explained by hypermethylation of the CpG island TET1 promoter region. Inactivation mutations in TET1 has not been observed in SI-NETs [4] suggesting further that other unknown mechanisms are responsible for lost expression.

In addition to the observed nuclear retention of TET2 during treatment of both SI-NET cell lines with leptomycin B, significant inhibition of cell proliferation and induction of apoptosis were observed. Thus, leptomycin B shows anti-cancer effects in SI-NET cells and suggests exportin-1 as a therapeutic target to restore the correct localization of tumor suppressors and other regulators of cancer growth. Leptomycin B showed toxicity and was not recommended for further clinical trials [33]. Since then, a new class of small molecule exportin-1 inhibitors has been developed; Selective Inhibitor of Nuclear Export (SINE) compounds. Importantly, the SINE compound KPT-330/selinexor showed the same anti-cancer effects as leptomycin B in SI-NET cells. Selinexor has shown promising results in phase I clinical studies in patients with multiple myeloma, advanced solid tumors, non-Hodgkin’s lymphoma, and acute myeloid leukemia [34–37]. A novel SINE compound (KPT-8602), with improved tolerability and that can be dosed daily, showed potent activity in vitro against acute lymphoblastic leukemia [38]. KPT-8602 is presently in human clinical trials (NCT02649790).

There are a few shortcomings in the present study. The limitations of sample size with only 40 patients and follow-up may result in finding no correlation between the level of 5hmC and patient’s clinical data. Also, in several tumor samples, consecutive sections were used for staining because the three antibodies specific for 5hmC, TET1 and TET2 shared the same host and hence double immunofluorescent staining was not possible to perform. In addition, this study did not further investigate mechanisms responsible for the loss of TET1 expression in SI-NETs, which could be investigated by chromatin immunoprecipitation experiments regarding a role of repressive histone modifications.

Based on the results presented here we suggest that SINE compounds such as KPT-330/selinexor or future developments should be further investigated with the aim of providing novel treatment options for SI-NET disease, either as single treatment or in combination with established treatment (somatostatin analogues, peptide receptor radiotherapy and everolimus) [39, 40].

## Conclusions

Loss of 5hmC and decreased expression of TET1 and TET2 have been associated with various types of human malignancies. Here we observed aberrant expression of TET1 and TET2, and variable levels of 5hmC in SI-NETs. Our results demonstrated a growth regulatory role of TET1 in vitro and loss of TET2 nuclear localization in SI-NETs. Treatment of SI-NET cells with leptomycin B and KPT330/selinexor, both inhibitors of exportin-1, suggested involvement of the exportin-1 nuclear export machinery in aberrant partitioning of TET2. These novel findings suggest that KPT-330/selinexor should be considered and evaluated for potential treatments in patients with SI-NET disease.

## Additional files

Additional file 1: **Table S1.** Clinical data for patients with SI-NETs. (PDF 1148 kb)

Additional file 2: **Figure S1.** IHC analysis of synaptophysin, CD45, and CD3 in CNDT2.5 and KRJ-I cells. **a**: Both CNDT2.5 and KRJ-I cells show positive staining for synaptophysin. Scale bar, 50 μm. **b**: Negative staining of KRJ-I cells for CD45 and CD3. Scale bar, 50 μm. Two SI-NETs with clusters of lymphoid cells were used as positive controls. Scale bar, 100 μm. (TIF 5235 kb)

Additional file 3: **Figure S2.** IHC analysis of 5hmC, TET1, and TET2 in normal small intestine. Scale bar, 50 μm. Staining is seen in chromogranin A positive cells. These cells likely represent the enterochromaffin cell of origin of SI-NETs. (TIF 4968 kb)

Additional file 4: **Figure S3.** DNA methylation analysis of 12 CpGs by quantitative bisulfite pyrosequencing. Down-regulation of TET1 expression in SI-NETs is not due to promoter hypermethylation. **a**: Effects on TET1 and TET2 mRNA expression after inhibition of DNA methylation by 5-aza-2′-deoxycytidine (Aza). *, *p* < 0.05. **b**: Quantitative bisulfite pyrosequencing analysis of 12 CpG residues in the CpG island TET1 promoter and exon 1 region. Cell lines CNDT2.5 and KRJ-I, and PTs and Mets (*n* = 20) are analyzed. (TIF 903 kb)

## Abbreviations

5hmC: 5-hydroxymethylcytosine
Aza: 5-aza-2′-deoxycytidine
CDKN1B: Cyclin-dependent kinase inhibitor 1B
cDNA: Complementary DNA
CRISPR: Clustered regularly interspaced short palindromic repeats
CTNNB1: Catenin beta 1
DAB: 3,3′-diaminobenzidine
EDTA: Ethylene diamine tetra acetic acid
ELISA: Enzyme-linked immunosorbent assay
GAPDH: Glyceraldehyde-3-phosphate dehydrogenase
HCl: Hydro chloric acid
HRP: Horse radish peroxidase
IHC: Immunohistochemistry
Met: Metastasis
mRNA: Messenger RNA
NaOH: Sodium hydroxide
NIH: National institutes of health
PBS: Phosphate buffered saline
PEST: Penicillin-streptomycin
PT: Primary tumor
RASSF1A: Ras association domain family member 1
rRNA: Ribosomal RNA
RT-PCR: Reverse transcription polymerase chain reaction
shRNA: Short hairpin RNA
SINE: Selective inhibitor of nuclear export
SI-NET: Small intestinal neuroendocrine tumor
TCEB3C: Transcription elongation factor B, polypeptide 3C
TET: Ten-eleven translocation
UV: Ultraviolet
XPO1/CRM1 exportin-1: Chromosome region maintenance 1
