# Supplementary material for: Decrease of 5-hydroxymethylcytosine and TET1 with nuclear exclusion of TET2 in small intestinal neuroendocrine tumors
Source: BMC Cancer. 2018 Jul 25;18:764. doi: 10.1186/s12885-018-4579-z (PMC6060499; doi:10.1186/s12885-018-4579-z)
Supplement: Supplementary file 1 — Table S1. Clinical data for patients with SI-NETs. (PDF 1148 kb) [file 12885_2018_4579_MOESM1_ESM.pdf]

Table S1. Clinical data for patients with SI-NETs

Patient no.	Gender	Liver tumor load *	Extrahepatic metastases	Peritoneal carcinomatosis	Carcimiod heart disease	U-5-HIAA	CGA	Ki67 at diagnosis (%)	ENETS grading	Survival (follow up years)	Status	SSA *	PRRT *	Interferon	Stage at diagnosis	Liver surgery	Liver metastases *
1	Female	2	Yes	No	Yes	28	NA	1	1	2.7	DWD	1	0	0	4	1	1
2	Male	0	No	Yes	No	84	18.3	3	2	5.1	AWD	1	0	0	4	0	1
3	Male	2	No	No	No	405	NA	2	1	3.5	AWD	1	0	0	4	1	1
4	Male	0	No	No	No	41	4.6	1	1	2.8	AWD	1	0	0	3b	0	2
5	Male	2	Yes	No	No	165	47	1	1	2.3	AWD	1	1	0	4	0	1
6	Male	0	No	No	No	66	9.1	2	1	1	AWD	1	0	0	4	1	1
7	Male	3	No	Yes	No	468	23	1	1	5.5	AWD	1	0	0	4	0	1
8	Female	0	No	No	No	NA	NA	7	2	6.8	DWD	1	0	0	3b	0	0
9	Female	0	No	No	No	NA	NA	1	1	3.3	AWOD	1	0	0	3b	0	0
10	Male	3	No	No	Yes	942	NA	1	1	3.3	AWD	1	0	0	4	1	1
11	Male	3	No	No	No	1564	NA	4	2	3.2	AWD	1	1	0	4	0	1
12	Male	0	No	No	No	117	6.3	2	1	3.2	AWD	1	0	0	4	0	0
13	Female	2	No	No	No	306	NA	1	1	7.8	AWD	1	0	1	4	1	1
14	Female	3	No	No	No	121.5	7.7	10	2	12	AWD	1	0	1	4	0	1
15	Female	2	No	Yes	No	NA	NA	1	1	8.8	DWD	1	0	1	4	0	1
16	Female	3	No	No	Yes	809	107	1	1	18.5	AWD	1	1	1	4	0	1
17	Female	2	No	No	No	NA	NA	1	1	11	AWD	1	0	1	4	1	1
18	Male	1	No	No	No	80	11.7	5	2	8.6	DWD	1	0	0	4	1	1
19	Female	3	No	Yes	No	1063	205	7	2	4.3	DWD	1	1	1	4	0	1
20	Female	1	No	No	No	74.4	10.6	5	2	15.4	AWD	1	0	1	4	1	1
21	Male	3	No	Yes	No	2400	NA	2	1	0.6	DWD	0	0	1	3b	0	0
22	Female	3	Yes	Yes	Yes	876	226	1	1	4.9	AWD	1	0	1	4	0	1
23	Female	3	No	No	No	NA	NA	1	1	14.8	DWD	1	0	1	4	0	1
24	Male	2	No	Yes	No	87	NA	17	2	0.9	DWD	1	0	1	4	0	1
25	Female	0	No	No	No	NA	NA	2	1	29.9	DWD	1	0	1	3b	1	2
26	Male	3	No	No	No	192	NA	5	2	4.3	AWD	1	1	1	4	0	1
27	Male	0	No	No	No	59	2.7	1	1	3.2	AWD	1	0	0	3b	0	0
28	Male	0	No	Yes	No	104	7.4	3	1	3	AWD	1	0	0	4	0	2
29	Male	0	No	No	No	32	3	1	1	0.3	DWOD	1	0	0	3b	0	0
30	Female	0	No	No	No	33	3.9	3	2	0.6	AWD	1	0	0	3b	0	0
31	Male	0	No	No	No	47	4.8	1	1	1.3	AWOD	0	0	0	3b	0	0
32	Male	0	No	No	No	NA	NA	2	1	12.9	AWOD	1	0	1	3b	0	0
33	Female	3	Yes	No	No	NA	NA	1	1	13.1	DWD	1	0	1	4	0	1
34	Female	3	No	No	No	NA	NA	24	3	3.1	DWD	1	0	1	4	0	1
35	Female	3	No	No	No	134	14.5	3	2	10.4	AWD	1	0	1	4	1	1
36	Male	3	No	No	No	379	NA	2	1	10.1	AWD	1	0	1	4	0	1
37	Male	0	No	No	No	32	8.2	1	1	9.4	DWD	1	0	0	3b	0	2
38	Female	0	No	No	No	72	37	5	2	4.2	DWD	1	0	1	3b	0	2
39	Male	0	No	No	No	NA	NA	2	1	7.8	AWD	1	0	0	3b	0	0
40	Male	2	No	Yes	No	NA	NA	3	2	5.1	DWD	1	0	0	4	0	1

* Liver tumor load: <5 lesions in one liver lobe = 1; 5-10 liver lesions in both liver lobes = 2; >10 liver lesions = 3; no computed tomography = 4

NA = Not available

DWD dead with disease, DWOD dead without disease, AWD alive with disease, AWOD alive without disease

* SSA somatostatin analogue treatment

* PRRT peptide receptor radionuclide therapy

* Liver metastases: No = 0; synchronous = 1; metachronous = 2
