# Supplementary figures and images for: Decrease of 5-hydroxymethylcytosine and TET1 with nuclear exclusion of TET2 in small intestinal neuroendocrine tumors
Source: BMC Cancer. 2018 Jul 25;18:764. doi: 10.1186/s12885-018-4579-z (PMC6060499; doi:10.1186/s12885-018-4579-z)

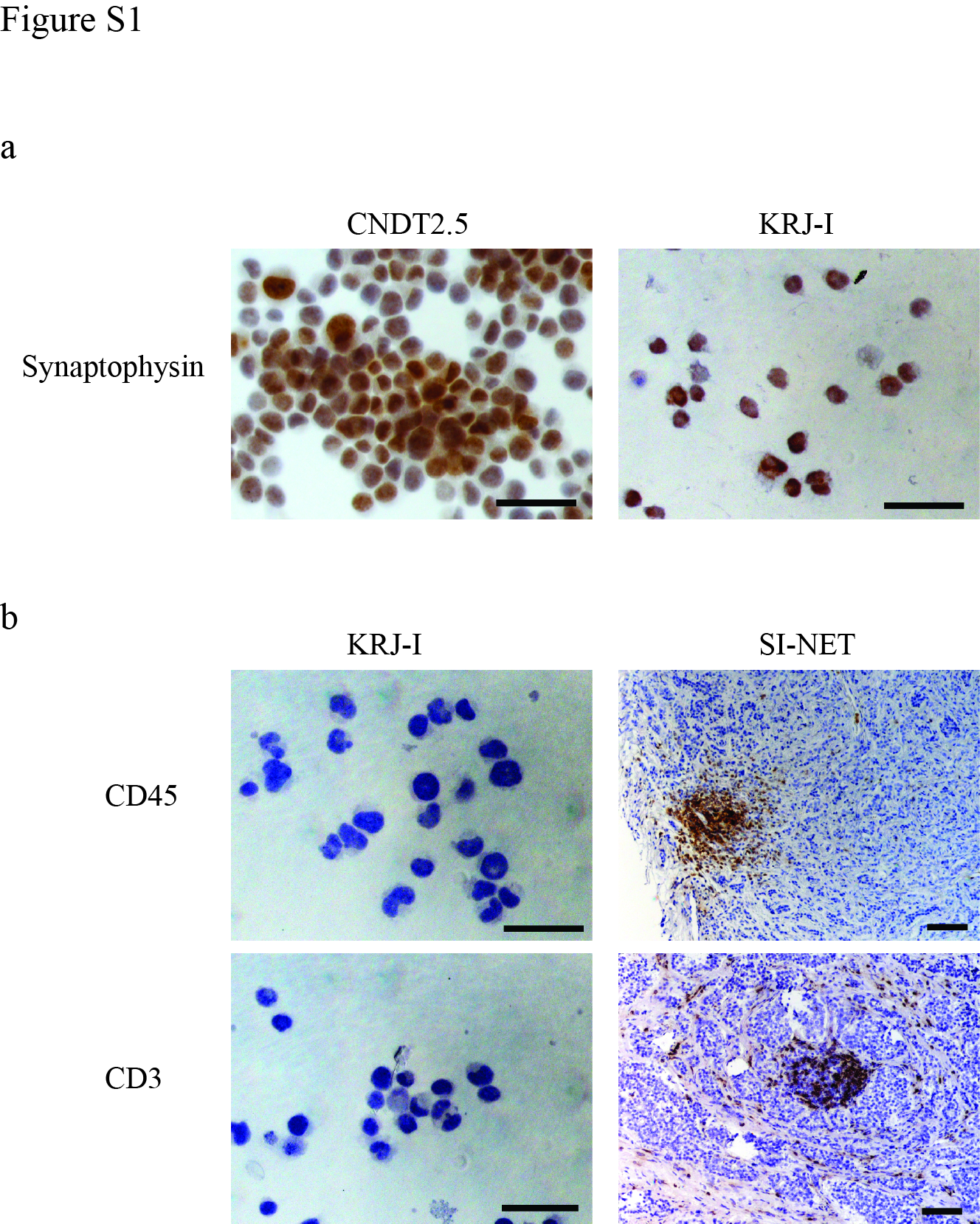

Supplement: Supplementary file 2 — Figure S1. IHC analysis of synaptophysin, CD45, and CD3 in CNDT2.5 and KRJ-I cells. a: Both CNDT2.5 and KRJ-I cells show positive staining for synaptophysin. Scale bar, 50 μm. b: Negative staining of KRJ-I cells for CD45 and CD3. Scale bar, 50 μm. Two SI-NETs with clusters of lymphoid cells were used as positive controls. Scale bar, 100 μm. (TIF 5235 kb) [file 12885_2018_4579_MOESM2_ESM.tif]

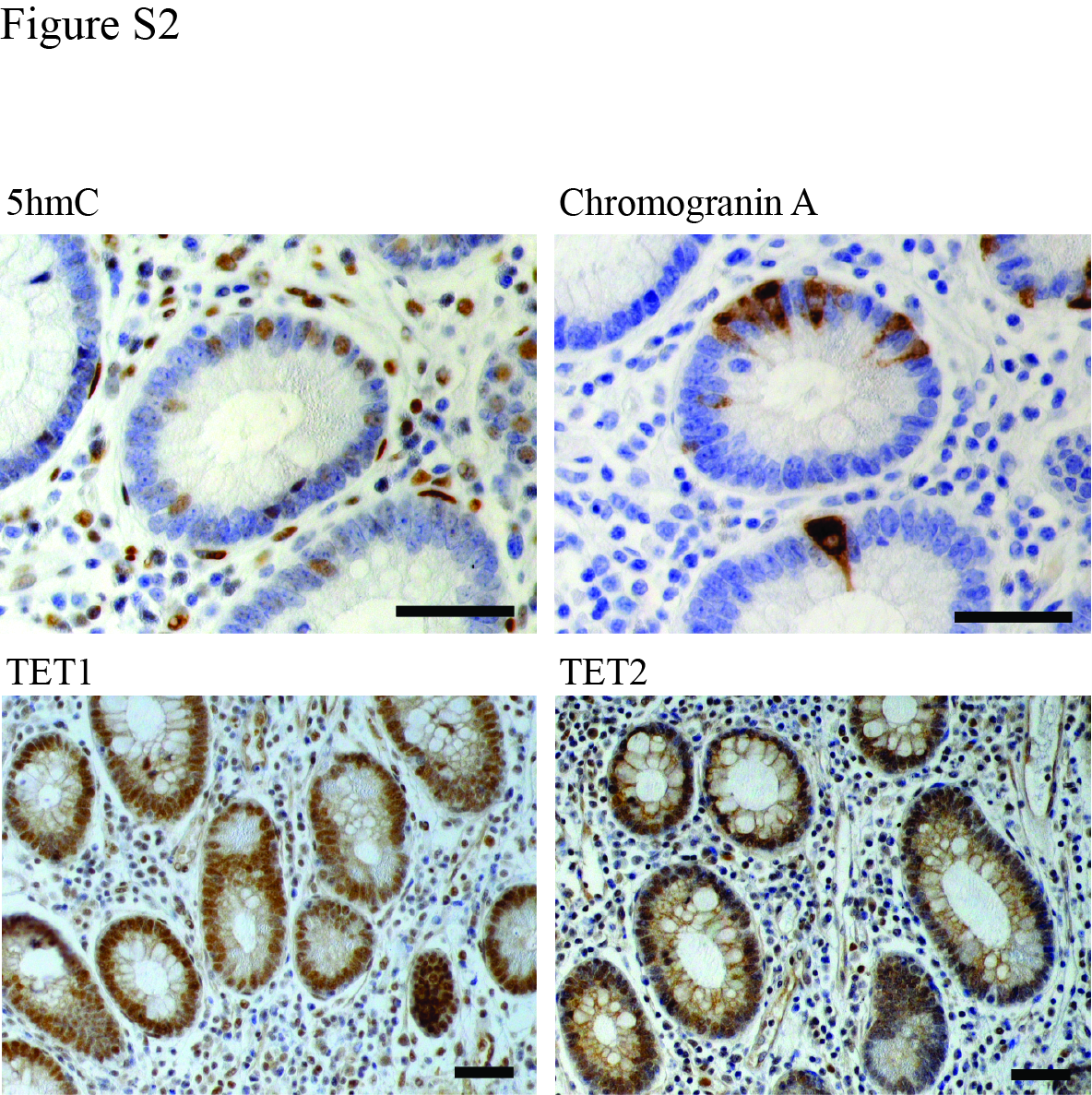

Supplement: Supplementary file 3 — Figure S2. IHC analysis of 5hmC, TET1, and TET2 in normal small intestine. Scale bar, 50 μm. Staining is seen in chromogranin A positive cells. These cells likely represent the enterochromaffin cell of origin of SI-NETs. (TIF 4968 kb) [file 12885_2018_4579_MOESM3_ESM.tif]

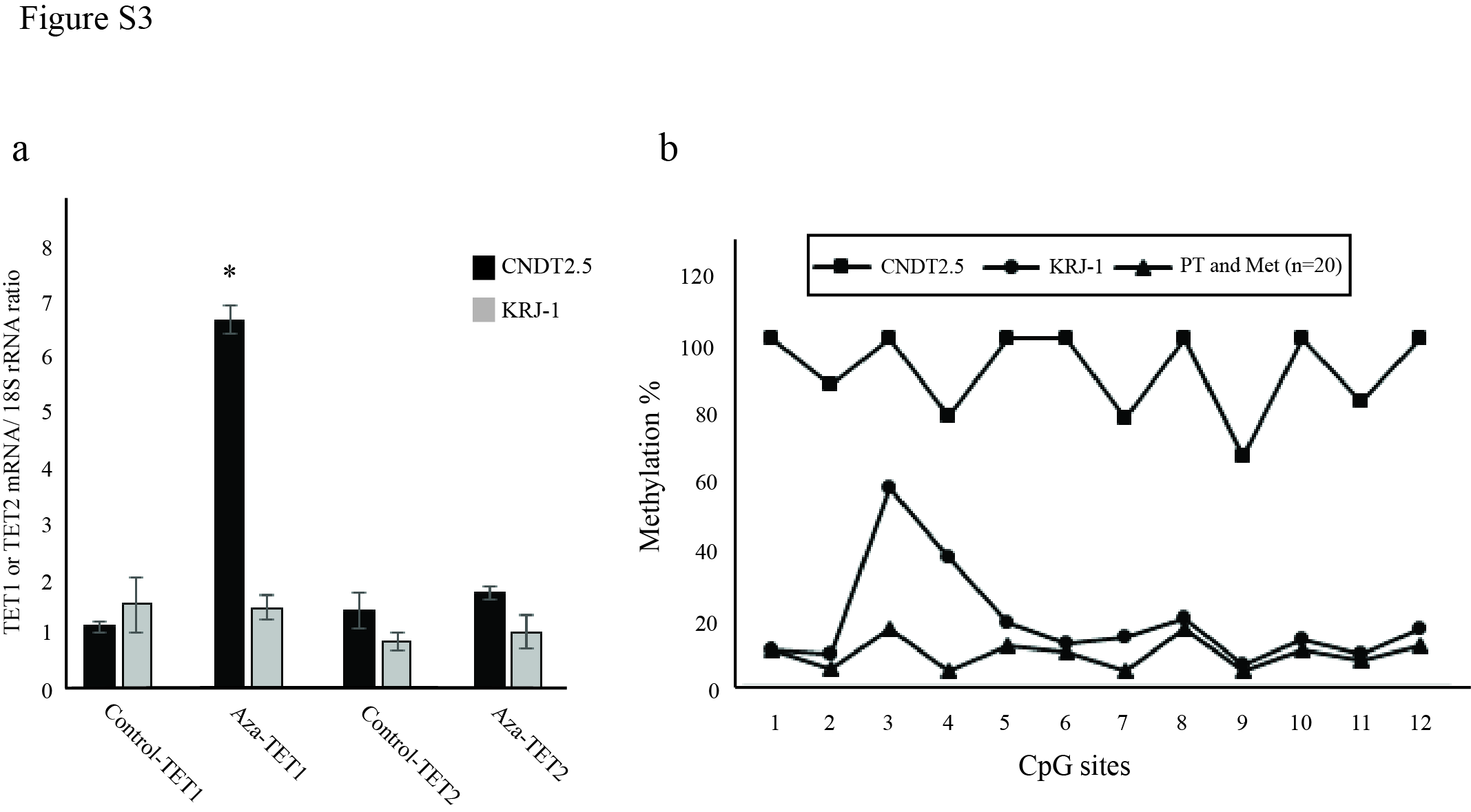

Supplement: Supplementary file 4 — Figure S3. DNA methylation analysis of 12 CpGs by quantitative bisulfite pyrosequencing. Down-regulation of TET1 expression in SI-NETs is not due to promoter hypermethylation. a: Effects on TET1 and TET2 mRNA expression after inhibition of DNA methylation by 5-aza-2′-deoxycytidine (Aza). *, p < 0.05. b: Quantitative bisulfite pyrosequencing analysis of 12 CpG residues in the CpG island TET1 promoter and exon 1 region. Cell lines CNDT2.5 and KRJ-I, and PTs and Mets (n = 20) are analyzed. (TIF 903 kb) [file 12885_2018_4579_MOESM4_ESM.tif]
